# Remarks on the Apothecaries' Act

**Published:** 1816-01

**Authors:** 


*For the London Medical and Physical Journal.*

*Remarks on the Apothecaries' Act;* by a SURGEON AND APOTHECARY.

PERHAPS some of your intelligent correspondents will favour your numerous readers with an explanation of that clause in the Apothecaries' Act which refers to druggists. The words are—"Provided always, and be it further enacted, that nothing in this Act contained shall extend, or be construed to extend, to prejudice, or in any way to affect the trade or business of a chemist and druggist, in the buying, preparing, compounding, dispensing, and vending drugs, medicines, and medicinal compounds, wholesale and retail; but all persons using or exercising the said trade or business, or who shall or may hereafter use or exercise the same, shall and may use, exercise, and carry on the same trade or business, in such manner, and as fully and amply to all intents and purposes, as the same trade or business was used, exercised, or carried on by chemists and druggists before the passing of this Act."

These gentlemen, under a supposition of being sheltered by the above clause, continue to exercise the various functions of the surgeon, the apothecary, and even the midwife. Patients are not only prescribed for at their shops, but regularly visited at their houses, by men who have never received any further education than that which has been obtained behind a druggist's counter, or extracted from such

 valuable valuable and instructive works as "Culpepper's Family Herbal," or Reeve's "Medical Guide."

Now, if the first clause in the Act, which I have transcribed below,* has any meaning, can you suppose that a man merely engaged in the trade or business of a chemist and druggist, that is, buying, preparing, compounding, dispensing, and vending drugs, &c. wholesale and retail, is protected by the clause alluded to, in thus trifling with the lives of his fellow creatures?

*London; Nov.* 10, 1815.

---

Our correspondent should remark, that the Act takes no notice of those now in practice. For the future, we conceive chemists and druggists will be prevented from visiting from home; but we must admit that the Act, in common with most others, is obscure in some parts.—Edit.

---

### COLLECTANEA MEDICA,

* "That from and after the first day of August, 1815, it shall not be lawful for any person or persons (except persons already in practice as such), to practise as an apothecary in any part of England or Wales, unless he or they shall have been examined by the Court of Examiners, or the major part of them, and have received a certificate of his or their being duly qualified to practise as such, from the said Court of Examiners, who are authorised and required to examine all person or persons applying to them, for the purpose of ascertaining the skill and abilities of such person or persons in the science and practice of medicine, and his or their fitness and qualification to practise as an apothecary."

